# Identification of an anti-plant-virus molecule in *Alpinia zerumbet*

**DOI:** 10.1186/s40643-021-00371-9

**Published:** 2021-02-21

**Authors:** Tadashi Hatanaka, Mari Narusaka, Misugi Uraji, Yasuyuki Yamaji, Yoshihiro Narusaka

**Affiliations:** 1 Okayama Prefectural Technology Center for Agriculture, Forestry, and Fisheries, Research Institute for Biological Sciences (RIBS), 7549-1 Kibichuo-cho, Kaga-gun, Okayama, 716-1241 Japan; 2 Present Address: Graduate School of Science, Technology and Innovation, Kobe University, 1-1, Rokkodai-cho, Nada-ku, Kobe, 657-8501 Japan; 3 Department of Agricultural and Environmental Biology, Graduate School of Agricultural and Life Sciences, The University of Tokyo, 1-1-1, Yayoi, Bunkyo-ku, Tokyo, 113-8657 Japan

**Keywords:** *Alpinia zerumbet*, Shell ginger, Proanthocyanidin, Anti-plant-virus molecule

## Abstract

In plants, viral diseases are second only to fungal diseases in terms of occurrence, and cause substantial damage to agricultural crops. The aqueous extracts of shell ginger, *Alpinia zerumbet* exhibit inhibitory effects against virus infections in belonging to the Solanaceae family. In this study, we isolated an anti-plant-virus molecule from the extracts using a conventional method involving a combination of reversed phase column chromatography, dialysis, and lyophilization. The anti-plant-virus molecule was identified as proanthocyanidin, which mostly consisted of epicatechin and exhibited more than 40 degrees of polymerization.
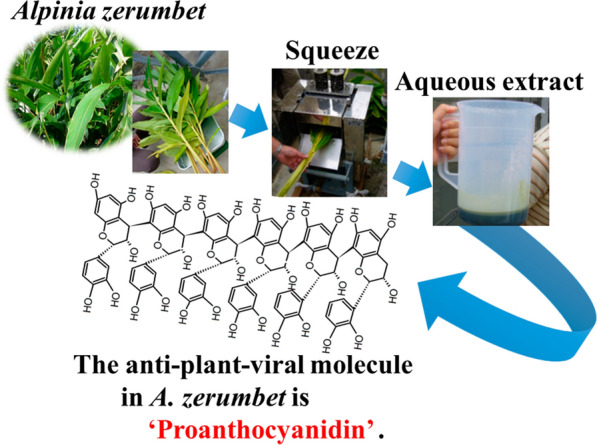

## Introduction

Recent advances in biotechnology and chemistry of natural products have enabled to make substantial progress in bio-pesticide development. Bio-pesticides have garnered considerable interest in recent years because they are environmentally safe, owing to the fact that they decompose quickly and leave little residue. The production of bio-pesticides is currently growing at a rate of 16% per year, which is approximately three-times higher than that of conventional agrochemicals (5.5% per year, Marrone 2013).

In plants, viral diseases are second only to fungal diseases in terms of occurrence, causing substantial damage to agricultural crops. Globally, plant viruses cause economic losses of approximately $60 billion annually, and the loss of food crops alone account for $20 billion per year (Xie et al. 2009).

It is well known that plant viruses are difficult to control compared to other plant pathogens primarily due to the lack of chemicals that are effective against plant viruses. To limit the damage caused by plant viruses, different management strategies have been applied, including prevention of plant virus spread by limiting the spread of transmitting vectors using insecticides, breeding cultivars that are resistant to virus infection, or simply cutting down infected plants. However, these efforts are still limited, and are not effective enough. Researchers have attempted to identify and develop anti-plant-virus agents, and recently, significant progress has been made in the field of biogenic anti-plant-virus substances (Zhao et al. 2017). Biogenic anti-plant-virus substances include proteins, polysaccharides, and small molecules from plants, microorganisms, algae, and animals. However, the number of anti-plant-virus substances with high efficiency and good economic benefits is still limited (Zhao et al. 2017).

More than 500 compounds have been isolated from 35 *Alpinia* species, and the major compounds are terpenoids and diarylheptanoids. The crude extracts and identified compounds exhibit a broad spectrum of bioactivities including anti-emetic, anti-ulcer, anti-bacterial, anti-inflammatory, anti-amnesic, and anti-cancer activities (Ma et al. 2017). Shell ginger, *A. zerumbet* (Pers.) B.L. Burtt & R.M. Smith, a member of the Zingiberaceae family (Teschke and Xuan 2018), grows widely in subtropical and tropical regions in East Asia, including the Okinawa Islands in Japan*. A. zerumbet* is commonly used in traditional Okinawa cuisine and as a herbal medicine. Bioactive compounds have been isolated and identified from crude *A. zerumbet* extracts (Tawata et al. 2008). A recent study demonstrated that *A. zerumbet* substantially increased the lifespan of *Caenorhabditis elegans* (Upadhyay et al. 2013).

In our previous study, we found that aqueous extracts of *A. zerumbet* exerted inhibitory effects against virus infection in plants belonging to the Solanaceae family (Narusaka et al. 2020).

In the present study, we isolated and identified an anti-plant-virus molecule, proanthocyanidin (PAC), in *A. zerumbet*.

## Materials and methods

### Chemicals

Procyanidins A2 and B1 were purchased from Funakoshi (Tokyo, Japan). 4-Dimethylaminocinnamaldehyde (DMAC) was obtained from Sigma-Aldrich (Tokyo, Japan).

### Plant material

We collected *A. zerumbet* plants, known as “*Gettou*” or “*Shima*-*gettou*”, in Nakijin Village, Okinawa Prefecture, Japan, and used the extracts of these plants to identify anti-plant-viral molecules.

### Purification of the anti-plant-viral molecule

An aqueous extract of *A. zerumbet* was obtained by squeezing the leaves and stems using a sugar cane squeezer (YBK-2, Yabiku, Okinawa, Japan). The extract was centrifuged (3260×*g*) for 10 min, and the supernatant was filtered to remove debris. Filtrate (100 mL) was applied to a C18 Sep-Pak® cartridge (10 g (35 cc); Nihon-Waters, Tokyo, Japan), which was equilibrated with distilled water before use. Thereafter, the column was washed with 100 mL of distilled water and eluted with 100 mL of 5%, 10%, 20%, and 40% acetonitrile. The fraction rich in anti-plant-viral molecules was obtained by elution with 100 mL of 20% acetonitrile. The fraction was evaporated to remove acetonitrile under reduced pressure at 50 °C, and the resultant solution was dialyzed using a dialysis membrane (cutoff 10 kDa) against distilled water (4 L × 4 times), and the dialysate was lyophilized. The resultant powder was used to characterize the purified anti-plant-viral molecule*.* The anti-plant-viral molecule was characterized by matrix-assisted laser desorption/ionization time of flight (MALDI-TOF) mass spectrometry (MS), ^13^C nuclear magnetic resonance (NMR) analysis, gel permeation chromatography (GPC), and spectrophotometric assays using DMAC.

### Antiviral assay

The antiviral assay was performed following a method described in our previous study (Narusaka et al. 2020). To evaluate the inhibitory effect of purified sample on viruses, the sample solution was applied on *Nicotiana benthamiana* plants (in the third true leaf stage) as foliar sprays only once; three days post treatment, the plants were inoculated with tomato mosaic virus (ToMV). Inoculation of *N. benthamiana* plants with ToMV was performed as described, with some modifications, including using pTL-derived plasmids (pTLBN.G3), which contain a full-length ToMV cDNA and a gene encoding green fluorescent protein (GFP) (Kubota et al. 2003). In vitro transcription of 2 μg of the template DNA using the AmpliCap-Max T7 High Yield Message Maker Kit (CELLSCRIPT, Madison, WI, USA) was performed at 37 °C for 40 min in a 20-μL reaction. Third true leaves of *N. benthamiana* that were treated with either the sample solution or distilled water (controls) were mechanically inoculated with 20 μL of 40-fold dilution of the transcription mixture*.* GFP foci were used to detect virus infection, and they were observed under blue light irradiation at 3 days post-inoculation (dpi).

The antiviral activity was assessed based on the number of GFP foci formed on the control and the treated *N. benthamiana* leaves. Statistical analyses were performed using one-way analysis of variance (ANOVA) with the Tukey–Kramer method.

### MALDI-TOF-MS analysis

The purified sample (1 mg) was dissolved in 1 mL of distilled water. 2,5-dihydroxy benzoic acid was used as a matrix in the positive-ion mode. To enhance ion formation, sodium chloride was added to the matrix. A MALDI-TOF-MS spectrum was acquired using a Shimadzu MALDI-8020 MALDI-TOF-MS apparatus (Shimadzu, Kyoto, Japan).

### DMAC assay

We performed DMAC assays as previously described by Wang (Wang et al. 2016). A methanol-based DMAC reagent was prepared by adding 50 mg of DMAC to 17.5 mL of hydrochloric acid (37%), and then making up the volume to 50 mL with methanol. The purified sample stock solution (2.5 mg/mL) was diluted with methanol to 0.25 mg/mL. A mixture of 0.125 mL of sample solution and 0.875 mL of DMAC reagent was prepared in cuvettes, with a 1-cm pass length. The spectrum (400–700 nm) of the DMAC conjugate was measured using an SH-8000 spectrometer (CORONA Electric, Hitachi, Japan).

### GPC analysis

The GPC analysis was performed at Toray Research Center (Tokyo, Japan). The purified sample (1 mg) was dissolved in 1 mL of dimethyl formamide (DMF) containing 50 mM lithium chloride (LiCl), and 200 μL of the sample was subjected to GPC. The molecular mass was estimated using a high-performance liquid chromatograph equipped with a refractive index detector (RI-8020; TOSOH, Tokyo, Japan), and TSK gel α-4000 and TSK gel α-2500 columns (7.8 mm × 30 cm; TOSOH, Tokyo, Japan), which were connected directly. DMF containing 50 mM LiCl was used as the solvent at a flow rate of 0.7 mL/min and the column temperature was maintained at 23 °C. The eluted sample was used for molecular mass determination, with a calibration curve obtained using standard polystyrene kits (PSt Quick E and F; TOSOH, Tokyo, Japan).

### ^***13***^***C-NMR analysis***

We used a Varian VNMRS (600 MHz) spectrometer (Varian, Palo Alto, CA, USA) for the analysis. The purified sample was dissolved in acetone-d6/D_2_O at a ratio of 1:1.

## Results and discussion

### Purification of an antiviral molecule in *A. zerumbet*

**Fig. 1:**
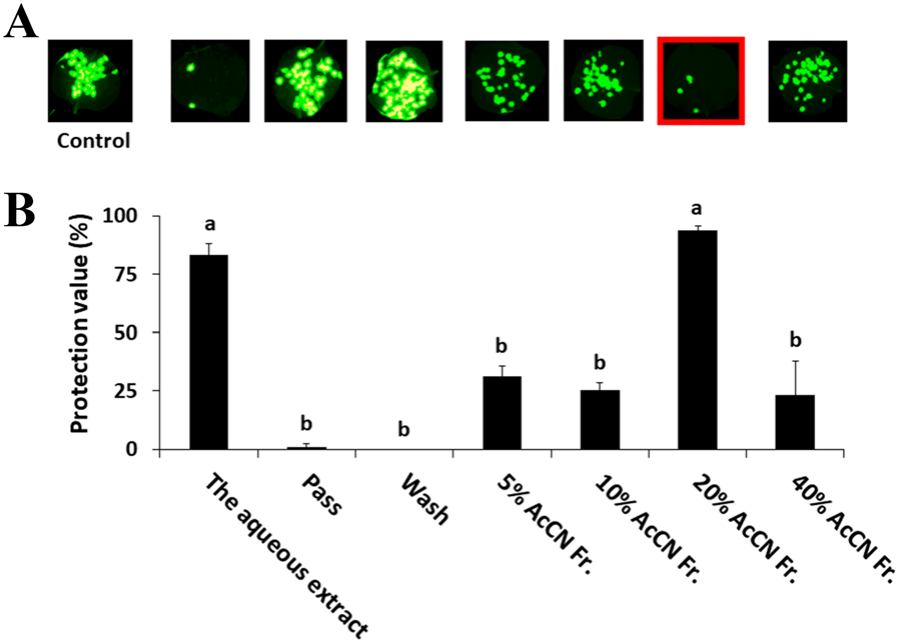
Antiviral activities of fractions of *A. zerumbet* extract separated by reverse-phase chromatography. The concentration of each fraction used in the application of samples to *N. benthamiana* leaves was adjusted to be the concentration of the original solution. *N. benthamiana* leaves were inoculated with GFP-tagged ToMV, and treated with extract samples or fractions separated by a C18 Sep-Pak® cartridge. **A** GFP foci formed at 3 days post-inoculation (dpi). Images were taken under blue light irradiation using the ChemiDoc™ MP Imaging System (Bio-rad, Hercules, CA, USA). **B** The GFP spots formed on the inoculated leaves were counted at 3 dpi. Protective value (%) = (1 − number of GFP spots on the treated plants/number of GFP spots on the control plants) × 100. Different letters (a and b) indicate significant differences between the control and treated plants at each leaf position by one-way ANOVA with the Tukey–Kramer method (*p* < 0.05)

**Fig. 2:**
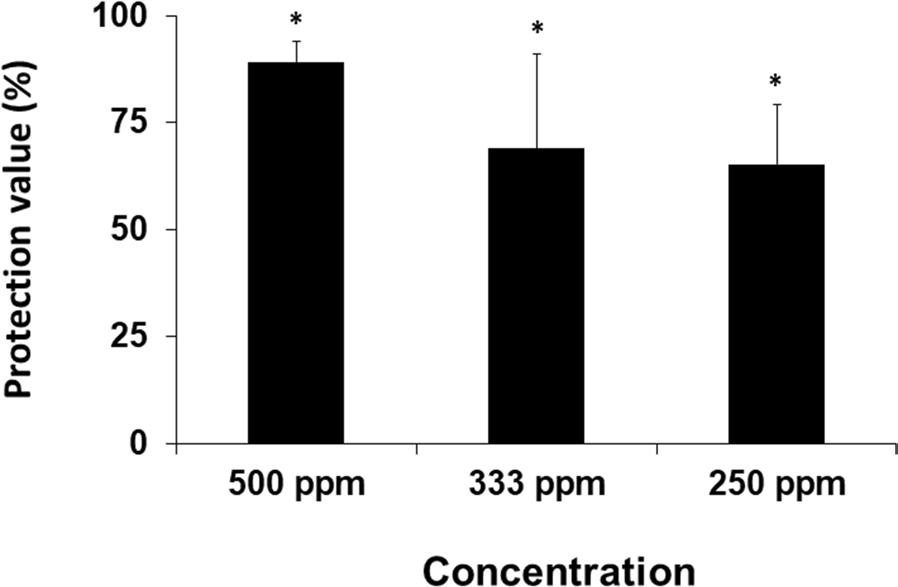
Antiviral activities of purified *A. zerumbet* extracts against GFP-tagged ToMV virions. *Nicotiana benthamiana* plants were treated with water (control) and different concentrations (250 ppm to 500 ppm) of purified samples from the aqueous extract of *A. zerumbet*, and then inoculated with GFP-tagged ToMV at three days post treatment. The GFP spots formed on the inoculated leaves were counted at 3 dpi. Protecting value (%) = (1 − number of GFP spots on the treated plants/number of GFP spots on the control plants) × 100. Asterisks indicate significant differences between the control and treated plants at each leaf position by one-way ANOVA with the Tukey–Kramer method (*p* < 0.05)

When the aqueous extract of *A. zerumbet* was fractioned using a reverse-phase chromatography and an antiviral assay was performed (Fig. 1), we found that the antiviral molecule was concentrated in the fraction obtained by elution with 20% acetonitrile. The fraction was then evaporated, dialyzed, and lyophilized. The resultant sample was used as the purified sample. The antiviral activity of the purified sample was confirmed by the inhibition against growth of GFP-tagged ToMV (Fig. 2). The protection value (%) of the purified sample increased as the final concentration of the sample applied to the leaves increased. Thus, we successfully purified an anti-plant-virus molecule from the aqueous extracts of *A. zerumbet* using a conventional method. To estimate the material balance in each fraction in the purification process, aliquots of each fraction were lyophilized (Table 1). The yield of the final fraction of the water extract was approximately 12% (w/v). This fraction was rich in the active molecule, accounting for ca. 10% (w/v) of the water extract of *A. zerumbet.*

**Table 1:** Material balance of each fraction in the purification process

Fraction	Lyophilized material (mg)	Yield (%)
Water extracts (100 mL)	4321	100.0
Pass and wash	3332	77.1
20% acetonitrile eluate	989	22.9
Dialysate	504	11.7

### MALDI-TOF-MS analysis

**Fig. 3:**
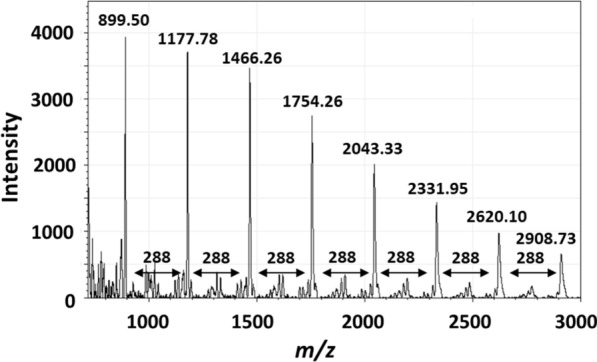
MALDI-TOF MS spectrum of the purified aqueous extract of *A. zerumbet*

As shown in Fig. 3, a series of peaks with ions corresponding to sodium adducts [M+Na^+^] distributed from *m/z* 889.5 to 6089.6 were observed in the mass spectrum of the purified sample obtained by MALDI-TOF-MS. The distance between the adjacent ion peaks was 288 Da (–C_15_H_12_O_6_–), corresponding to one catechin or epicatechin monomer. The results indicate that the active molecule is a polymer consisting of catechin or epicatechin. The mass intensity of the sample decreased with increasing degrees of polymerization (DP). The lower sensitivity of the large ions may be due to collisional fragmentations during the time of flight or when the ions are extracted from the matrix (Flamini 2003; Perret et al. 2003). MALDI-TOF-MS analysis enables the determination of the absolute molecular weight of individual chains with high accuracy, as long as the polymer is not too large (approximately < 10 kDa) (Koster et al. 2000). If intact molecular adducts are produced in the gas phase and the adducts are clearly identified, the analysis allows the confirmation of the nature of end-groups based on the sum of mass of adducted monomer units and both end-groups (Li et al. 2010; Charles 2014). From the data of the series of peaks of sodium adducts, we estimated the end structure of PAC. As shown in Table 2, both end structures were protons because the average molecular mass of both end-groups was 3.3 (ca. 2.0). Thus, the antiviral molecule in *A. zerumbet* is a PAC polymer, consisting of a simple condensation of catechin or epicatechin monomers.

**Table 2:** Estimation of end structures of PAC from *A. zerumbet*

Observed molecular mass (A)	Cation (Na^+^) molecular mass (B)	Exact molecular mass (A) − (B) = (C)	Unit molecular mass (D)	Degree of polymerization (C) ÷ (D) = (E)	Estimated end molecular masses (C) − ((D) × (E))
889.50	22.99	866.51	288.06	3	2.3
1177.78	22.99	1154.79	288.06	4	2.6
1466.26	22.99	1443.27	288.06	5	3.0
1754.55	22.99	1731.56	288.06	6	3.2
2043.43	22.99	2020.44	288.06	7	4.0
2332.06	22.99	2309.07	288.06	8	4.6
					Average
					3.3

### DMAC assay

**Fig. 4:**
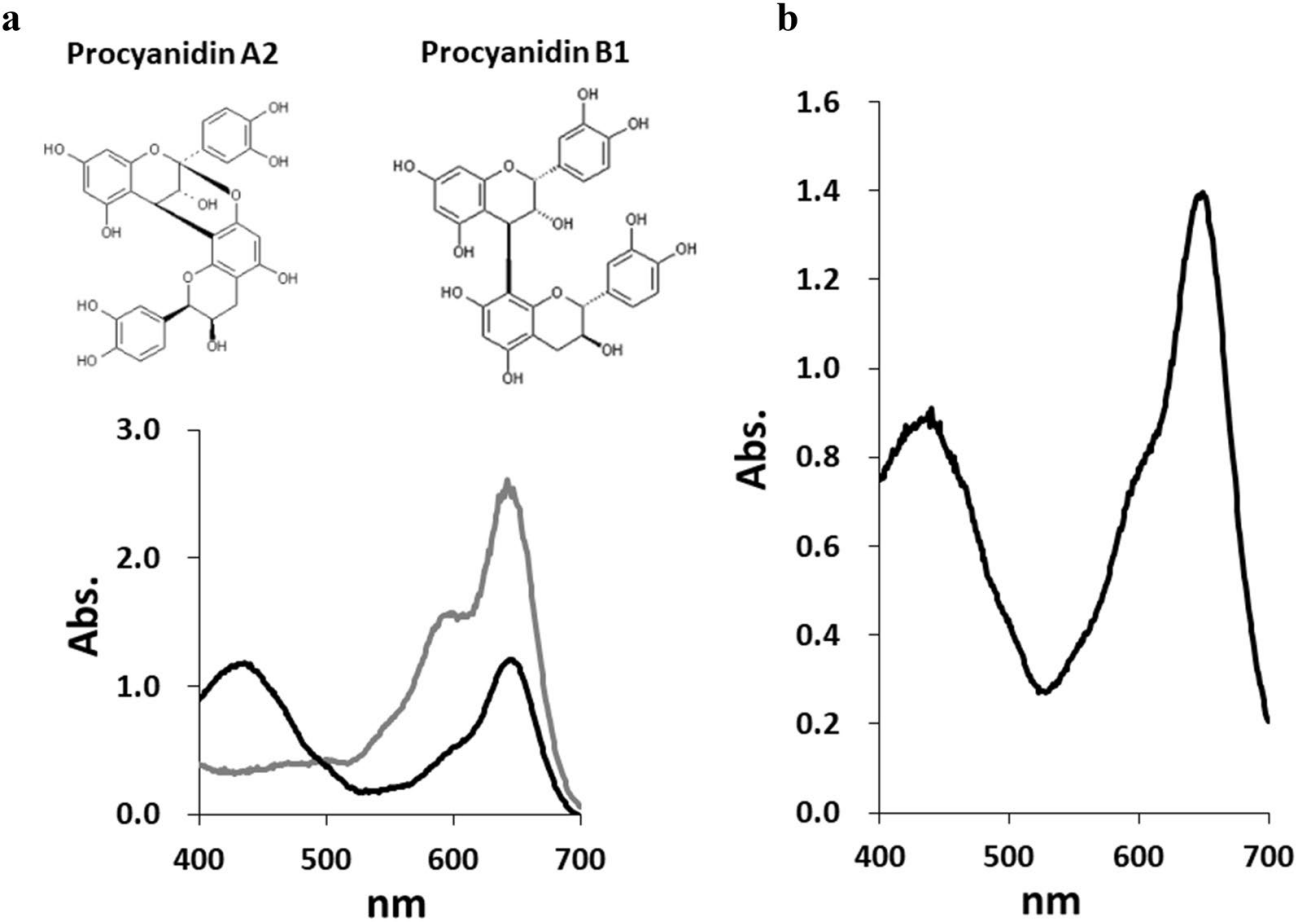
Visible spectra of procyanidins and the purified sample from *A.* zerumbet aqueous extracts. **a** Chemical structure of procyanidins A2 and B1 (upper), the spectra of procyanidin A2 (gray line) and procyanidin B1 (black line), determined using the DMAC assay (lower). **b** The spectrum of the purified sample from the aqueous extract of *A. zerumbet*, obtained using the assay

The polymeric nature of PACs causes in structural variations, including variations in the DP, linkage type, and position between constituent units. Catechin and epicatechin are the two most common flavan-3-ol units present in PAC polymers. The most common linkage of the flavan-3-ol units is a single C–C bond (B-type), between the C4 of one flavan-3-ol unit (referred to as upper) and C8 or C6 of another (lower) unit. Wang et al. reported that in the absorption spectra, B-type dimers including procyanidin B1 showed a secondary 440-nm absorption peak, in addition to the primary 640-nm peak, which is typically measured using the DMAC assay (Wang et al. 2016). The A-type dimmers, with double interflavan linkage, showed only a 640-nm absorbance peak in the assay (Fig. 4a). PAC in *A. zerumbet*-DMAC conjugate also showed a secondary 440-nm peak, as shown in Fig. 4b. Wang et al. have described that all B-type cocoa PACs exhibited a secondary 440-nm absorbance peak that declined in intensity when DP increased (Wang et al. 2016). Based on these results, the PAC from *A. zerumbet* was categorized as a B-type.

### GPC analysis

**Fig. 5:**
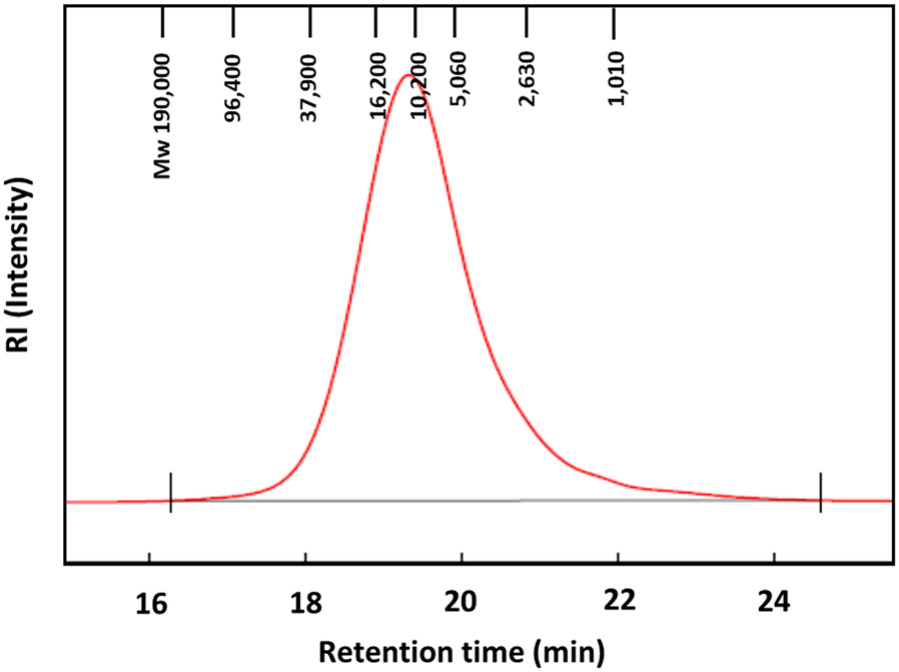
Molecular mass distribution of the purified sample from *A. zerumbet* aqueous extracts (red line). The red line indicates the molecular mass distribution

Figure 5 shows the molecular mass of PAC from *A. zerumbet*. The weight-average molecular weight (Mw), number average molecular weight (Mn), polydispersity (Mw/Mn), and DP of the PAC were 12,400, 5,640, 2.2, and 43, respectively.

### ^***13***^***C-NMR analysis***

**Fig. 6:**
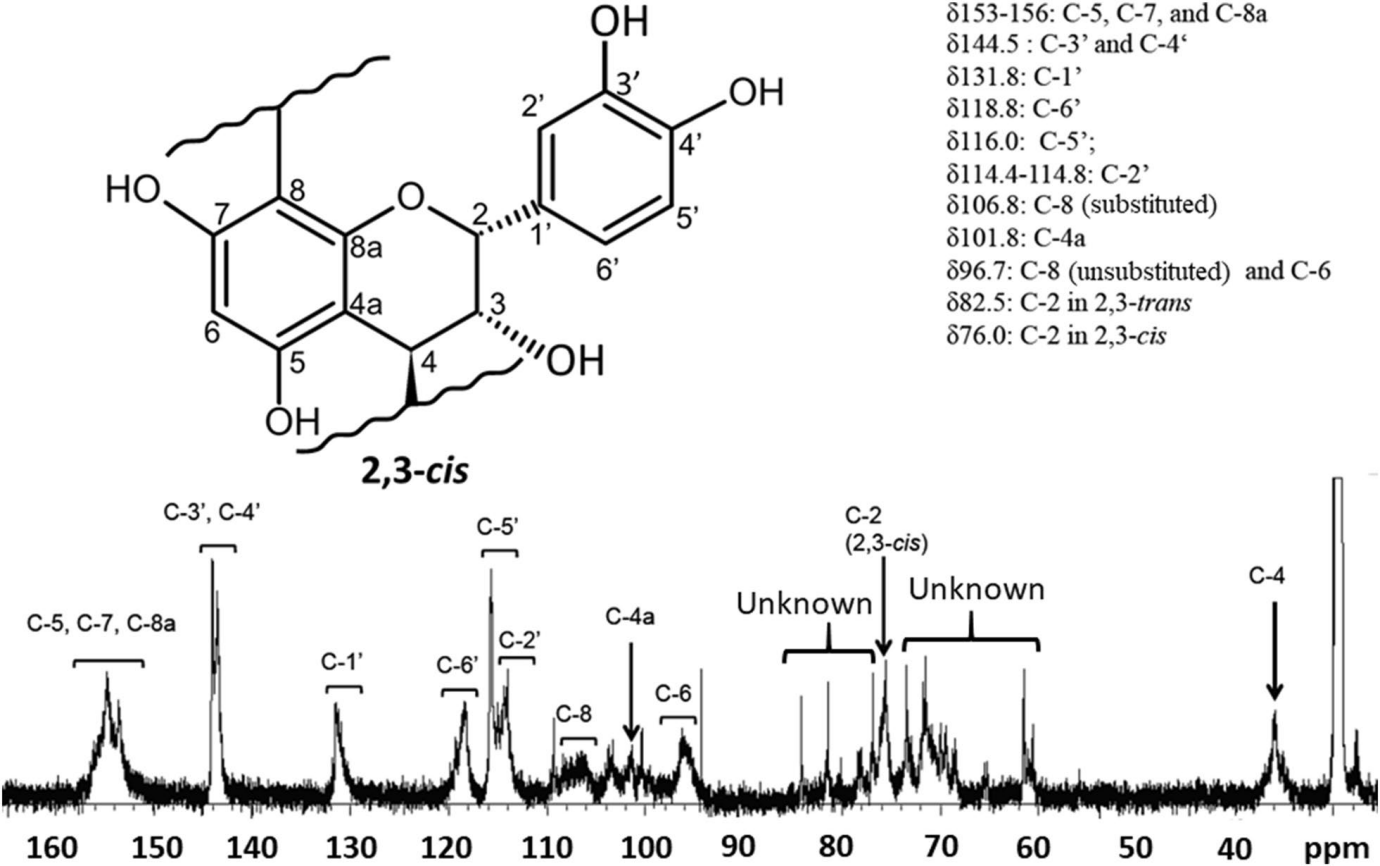
Solution-state ^13^C-NMR spectrum of proanthocyanidin polymer preparation of aqueous extracts of *A. zerumbet*

The spectrum of PAC from *A. zerumbet* is shown in Fig. 5. Resonance assignments were conducted according to a previous study (Eberhardt and Young 1994). All assignments were consistent with those reported by Eberhardt and Young (1994)*.* With respect to the aromatic B-ring attached at C-2, the orientation of the hydroxyl group at C-3 can be either cis or trans as shown in Fig. 6. The relative amounts of the two stereo-chemical configurations (2,3-cis or trans) in PAC from *A. zerumbet* were estimated from ^13^C-NMR spectra by integrating the resonances at 76.0 and 82.5 ppm corresponding to C-2 in 2,3-cis and 2,3-trans chain extender units, respectively. The results revealed that PAC in *A. zerumbet* almost consists of epicatechin (i.e., 2,3-cis configuration) because there was a low-intensity signal at 82.5 ppm, which was assigned a 2,3-trans configuration (Eberhardt and Young 1994). We observed several peaks between 68 and 80 ppm (Fig. 6) that could not be assigned, and these peaks may have been derived from impurities.

To the best of our knowledge, the present study is the first to report the identification of an anti-plant-virus molecule, PAC, in *A. zerumbet*. PACs are synthesized as polymeric end-products of flavan-3-ols (catechin or epicatechin), which are biosynthesized from phenylalanine and malonyl-CoA via the flavonoid pathway. Phenylalanine is derived from the shikimate pathway, and malonyl-CoA is obtained from citrate that is produced by the tricarboxylic acid cycle (Oliveira et al. 2013). However, the polymerization pathway of PACs remains unexplained. We are currently attempting to analyze the genome of *A. zerumbet* to elucidate biosynthesis pathway of the PAC described in this study. Further studies are necessary to determine the mechanism of anti-plant-virus activity of PAC.

## Conclusions

In this study, we identified the anti-plant-virus molecule in *A. zerumbet* as a PAC by using *Nicotiana benthamiana* plants and GFP-fused ToMV. The PAC, as the active molecule, was present at high proportions in the 10% (w/v) water extract. In addition, the PAC was categorized as B-type, with more than 40 DP, and almost consisted of epicatechin. Thus, *A. zerumbet* is a potential bioresource of pesticides against viral diseases in plants.

## Data Availability

All data generated or analyzed during this study are included in this published article.

## Abbreviations

DMAC: 4-Dimethylaminocinnamaldehyde
PAC: Proanthocyanidin
MALDI-TOF: Matrix-assisted laser desorption/ionization time of flight
MS: Mass
NMR: Nuclear magnetic resonance
GPC: Gel permeation chromatography
ToMV: Tomato mosaic virus
GFP: Green fluorescent protein
dpi: Days post-inoculation
ANOVA: Analysis of variance
DMF: Dimethyl formamide
LiCl: Lithium chloride
DP: Degree of polymerization
Mw: Weight-average molecular weight
Mn: Number average molecular weight
